# Tiered Protocol for Sussing Out Endocrine Disruption

**DOI:** 10.1289/ehp.121-a16

**Published:** 2013-01-01

**Authors:** Kellyn S. Betts

**Affiliations:** For more than a dozen years Kellyn S. Betts has written about environmental contaminants, hazards, and technology for solving environmental problems for publications including *EHP* and *Environmental Science & Technology*.

A team of experts in biology, chemistry, and toxicology has developed a protocol to help industrial scientists detect endocrine-disrupting tendencies early in the chemical development process.^1^ What sets the Tiered Protocol for Endocrine Disruption (TiPED) apart from the protocols typically used to assess product safety is its incorporation of what the authors believe to be the best assays for detecting effects on the endocrine system. TiPED offers industry what coauthor John Warner, president of the Warner Babcock Institute for Green Chemistry, calls an “à la carte palette of assays . . . that have been vetted by the environmental health community.”

The protocol was inspired by demand from consumers for safer materials, as well as from chemists and companies wishing to meet that demand, according to the authors. It starts with the fastest, simplest, cheapest tests and progresses to increasingly complex, expensive assays to identify endocrine activity—an issue the authors say is not currently addressed by U.S. chemical regulations. The authors aim to enable chemists to assess endocrine toxicity early in the design process when they are first developing new molecules, long before chemicals reach market. They expect the protocol to be used in addition to assays aimed at evaluating other toxicity end points.

Coauthor Pete Myers, CEO and chief scientist for the nonprofit Environmental Health Sciences, explains that the computational and receptor-based assays recommended in the first three tiers all make assumptions about the mechanism of endocrine disruption, whereas live-animal assays recommended in tiers 4 and 5 are designed to catch endocrine-disruption activity via mechanisms that are not yet identified. “If you’ve made it through a series of assays, all of which turn up negative, and the material has potential economic value, you will be more willing to do the more intensive and expensive assays,” he says.

**Figure:**
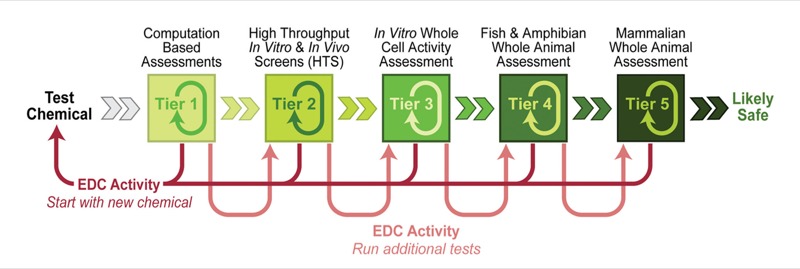
Failure to find endocrine-disrupting activity in one tier of TiPED (confirmed with other assays within the same tier) then leads to testing at the next highest tier. Chemists can begin testing at a tier that best fits their individual needs, depending on prior knowledge (or hypotheses) about potential mechanisms of action, as well as access to assay systems. Results from initial tests would then inform the next steps. Schug et al.; doi:10.1039/C2GC35055F

Chemicals that test positive for endocrine disruption early on can either be set aside without investing any further time or money, or potentially redesigned to eliminate this characteristic. The authors point out that BPA and phthalates, had they gone through TiPED, would have been identified as endocrine disruptors in Tier 1, while atrazine, perchlorate, and perfluorinated compounds might have made it to Tier 3 or 4 before being identified.^1^

The protocol appears “to be an effective systematic, progressive and pragmatic way for chemists to determine the potential endocrine-disrupting activity of a new chemical they are designing,” says Roger McFadden, senior scientist for consumer office goods retailer Staples, Inc. “Emerging domestic and international chemical regulations and a heightened consumer awareness about chemicals of concern in products is challenging businesses to take a more proactive and preemptive approach to supply chain management [and] materials selection at the product design stage,” he explains.

Market research firm Pike Research predicts the market for green chemistry will grow dramatically, from $2.8 billion in 2011 to $98.5 billion in 2020.^2^ The “clean little secret” that people in a wide variety of industries—including electronics, aerospace, cosmetics, agriculture, and energy—are increasingly recognizing is that green chemistry leads to good business decisions, says Paul Anastas, who directs Yale University’s Center for Green Chemistry and Green Engineering. Anastas made this comment at a meeting held in September 2011 by the National Academy of Sciences’ Standing Committee on Use of Emerging Science for Environmental Health Decisions, where scientists from DuPont, HP, and Pfizer talked about how green chemistry is influencing their businesses.^3^ Thaddeus Schug, the new paper’s lead author and a program administrator at the National Institute of Environmental Health Sciences, presented an earlier incarnation of TiPED at that meeting.

Pamela J. Spencer, associate director of product sustainability consulting for Dow Chemical Company’s Toxicology and Environmental Research and Consulting laboratory, points out that very few high-throughput screens cited in the publication are validated. Validation refers to the formal process through which assays are shown to dependably work as intended and thus produce reliable results that can be compared across studies.

Lack of validation does not necessarily mean an assay is invalid. But it does mean results from the assay could be considered unreliable by regulatory agencies, depending on other factors. However, Myers says TiPED “isn’t designed to inform regulation; it’s designed to provide [individual] chemists the knowledge base to develop a new generation of materials that are inherently safer.”

The authors envision that their protocol will change over time as new and improved assays are developed. “It’s a living protocol that can evolve with the science,” says coauthor Karen Peabody O’Brien, executive director of the nonprofit Advancing Green Chemistry. Additional information about the protocol is available at http://www.tipedinfo.com/.

Warner says he welcomes challenges to the protocol in the belief that they will eventually inspire the various factions to agree about what the right assays are. When that happens, the authors say that any business that follows the protocol can assure consumers, stockholders, and regulators that they made the best possible effort to ensure their new chemicals and products are not endocrine disruptors.
